# Decreased Total Iron Binding Capacity May Correlate with Ruptured Intracranial Aneurysms

**DOI:** 10.1038/s41598-019-42622-y

**Published:** 2019-04-15

**Authors:** Anil Can, Pui Man Rosalind Lai, Victor M. Castro, Sheng Yu, Dmitriy Dligach, Sean Finan, Vivian Gainer, Nancy A. Shadick, Guergana Savova, Shawn Murphy, Tianxi Cai, Scott T. Weiss, Rose Du

**Affiliations:** 1 Department of Neurosurgery, Brigham and Women’s Hospital, Harvard Medical School, Boston, MA USA; 2 Research Information Systems and Computing, Partners Healthcare, Boston, MA USA; 3 Center for Statistical Science, Tsinghua University, Beijing, China; 4 Department of Computer Science, Loyola University, Chicago, IL USA; 5 Boston Children’s Hospital Informatics Program, Boston, MA USA; 6 Division of Rheumatology, Immunology and Allergy, Brigham and Women’s Hospital, Boston, MA USA; 7 Department of Neurology, Massachusetts General Hospital, Boston, MA USA; 8 Biostatistics, Harvard School T. H. Chan of Public Health, Boston, MA USA; 9 Channing Division of Network Medicine, Brigham and Women’s Hospital, Boston, MA USA

## Abstract

Iron and its derivatives play a significant role in various physiological and biochemical pathways, and are influenced by a wide variety of inflammatory, infectious, and immunological disorders. We hypothesized that iron and its related factors play a role in intracranial aneurysm pathophysiology and investigated if serum iron values are associated with ruptured intracranial aneurysms. 4,701 patients with 6,411 intracranial aneurysms, including 1201 prospective patients, who were diagnosed at the Massachusetts General Hospital and Brigham and Women’s Hospital between 1990 and 2016 were evaluated. A total of 366 patients with available serum iron, ferritin and total iron binding capacity (TIBC) values were ultimately included in the analysis. 89% of included patients had anemia. Patients were categorized into ruptured and non-ruptured groups. Univariable and multivariable logistic regression analyses were performed to determine the association between ruptured aneurysms and iron, ferritin, and TIBC. TIBC values (10^−3^ g/L) within 1 year of diagnosis (OR 0.41, 95% CI 0.28–0.59) and between 1 and 3 years from diagnosis (OR 0.52, 95% CI 0.29–0.93) were significantly and inversely associated with intracranial aneurysm rupture. In contrast, serum iron and ferritin were not significant. In this case-control study, low TIBC was significantly associated with ruptured aneurysms, both in the short- and long term. However, this association may not apply to the general population as there may be a selection bias as iron studies were done in a subset of patients only.

## Introduction

Despite advances in the diagnosis and treatment of intracranial aneurysms, both morbidity and mortality of aneurysmal subarachnoid hemorrhage (aSAH) remain high^1,2^. There are two important issues in the treatment of patients with unruptured aneurysms. One is the determination of who should be treated aggressively vs conservatively and the other is the lack of a medical therapy that would reduce the risk of rupture in those who are treated conservatively. While there are already a number of criteria that clinicians typically use to determine the risk of rupture of an aneurysm, including aneurysm size^3^, morphology^4–6^, and location^4^, there are many patients who are in the “gray zone”, in whom a biomarker for risk stratification would be beneficial in determining the most optimal treatment paradigm. Second, while there are some modifiable risk factors such as smoking, alcohol use, and hypertension in those who are treated conservatively, there is currently no medical treatment that can reduce the risk of rupture. While we cannot infer causality with a biomarker association study, the identification of biomarkers that are associated with rupture may help suggest important causal pathways and motivate potential candidates for future studies. Iron and its reactive derivatives, major degradation products of hemoglobin and essential elements for numerous physiological, metabolic, and inflammatory pathways, are attractive candidates to investigate for their role in aneurysm pathophysiology. Although a few studies have investigated the effects of iron on cellular changes after subarachnoid hemorrhage, the relationship between iron and risk of intracranial aneurysm rupture is unknown^7–11^. Here we present a case-control study investigating the magnitude and direction of the association between rupture of intracranial aneurysms and iron values and its reactive derivatives. In order to control for the effects of acute-phase reactions after aneurysm rupture, we included both short-term and long-term measurements. Of note, the associations found in this study may be influenced by selection bias as iron studies were performed only in a subset of patients.

## Methods

With a combination of machine learning algorithms and manual chart review, we identified 4,701 patients who were diagnosed with an intracranial aneurysm at the Brigham and Women’s Hospital (BWH) and Massachusetts General Hospital (MGH) between 1990 and 2016^12^. This study was approved by the Partners Institutional Review Board and considered minimal risk, therefore informed consent was waived. All procedures performed were in accordance with the ethical standards of the institutional review board. We identified patients both prospectively on clinical presentation (2007–2016) and retrospectively using natural language processing (NLP) in conjunction with the Partners Healthcare Research Patients Data Registry (RPDR) which includes 4.2 million patients who have received care from BWH and MGH (1990–2013)^13^. We obtained an initial set of potential aneurysm patients from the RPDR with the use of ICD-9 and CPT codes, and we then used NLP to train a classification algorithm which yielded 5,589 patients^12^. 727 of these patients were also seen on clinical presentation from 2007–2013 with prospectively collected data^14^. We included 474 additional patients with prospectively collected data who were seen on clinical presentation from 2013–2016^14^. Then we reviewed the medical records and imaging studies of the 6,063 patients in detail (AC and RD) to ultimately identify 4,701 patients with definite saccular aneurysms, of which 1,302 (27.7%) presented with rupture^13^. Inclusion criteria were limited to patients with available serum iron, ferritin, and total iron binding capacity (TIBC) measurements within 1 year of diagnosis, leading to a final total number of 366 eligible patients. We also collected serum iron, ferritin, and total iron binding capacity levels between 1 and 3 years from diagnosis, for long-term analysis. Long-term measurements were available in 202 patients. We recorded the results of the imaging studies and excluded patients with possible infundibula or non-definitive diagnoses of aneurysms, feeding artery aneurysms associated with arteriovenous malformations, fusiform or dissecting aneurysms, and those lacking clinical notes or radiographic images^14^. We excluded patients who received treatment of their aneurysm(s) prior to presentation from this study and we categorized patients who presented with an aneurysmal subarachnoid hemorrhage as harboring a ruptured aneurysm^14^. Since patients were classified according to their rupture status at the time of diagnosis, patients with ruptured aneurysms who also had unruptured aneurysms were included and classified as ruptured. Patients were included if treatment occurred at a later point.

Patient characteristics including age, sex and race, and comorbidities including coronary artery disease (CAD), hypertension, myocardial infarction (MI), and atrial fibrillation (AF) were obtained^15^. A risk factor was assumed to be absent if we found no documentation of its presence^14^. In addition, we included the total number of intracranial aneurysms per patient, family history of aneurysms, and information on current tobacco and alcohol use. The diagnosis of aSAH was confirmed with a computed tomographic (CT) scan, cerebrospinal fluid analysis, or intraoperatively by a neurosurgeon^14^. Given the diffuseness of SAH, hemorrhage amount was determined via a modified Fisher grade where 1 is no SAH, 2 is ≤1 mm of SAH, and 3 is >1 mm of SAH. In patients with intracerebral hemorrhage (ICH), ICH volume was measured using the ABC/2 method^16^.

Differences in baseline characteristics between the ruptured and unruptured groups were evaluated using *t-*tests for continuous variables and Pearson’s chi-square test for categorical variables^6^. Univariable and multivariable logistic regression models were implemented to test for effects of serum iron, ferritin, and total iron binding capacity (TIBC) values with a backward elimination procedure to identify significant confounders. Cut-off p-values of 0.1 were used to select the set of variables to be included in the initial multivariable model for backward elimination^15^. Adjusted odds ratios (OR) with 95% confidence intervals (CI’s) were calculated, and p < 0.05 was considered significant^15^. Missing values were accounted for by using multiple imputation with chained equations and inferential statistics were obtained from 40 imputed datasets^12^. Sensitivity analysis using a subgroup consisting of complete cases only and males or females only was also performed. All statistical analyses were performed using the Stata statistical software package (version 14, StataCorp. College Station, TX)^14^.

## Results

Patient demographics and characteristics, as well as laboratory values, are shown in Table 1. 366 patients were included, of which 151 patients (41.3%) had ruptured aneurysms and 95 patients (26.0%) had low TIBC values (<2.20 10^−3^ g/L). In general, patients with low TIBC values were significantly more frequently white, less frequently black, and had lower iron and higher ferritin levels compared to patients without low TIBC values. In addition, patients with lower TIBC values were significantly more frequently diagnosed with ruptured aneurysms (72.6% vs. 30.3%, respectively). Table 2 shows iron, ferritin, TIBC, and albumin values stratified according to rupture status in both the short-term and long-term period. Ferritin was significantly lower among female patients compared to male patients (Supplemental Table 1), a finding which is in line with previous publications^17,18^. However, there was no sex difference in iron or TIBC. We evaluated the admission head CT of 139 out of 149 patients with ruptured aneurysms who had complete iron studies. 10 patients did not have imaging available for review. Of the 139 patients, there were 20 who also had an intracerebral hemorrhage. There was no association between TIBC and the modified Fisher grade (β = 1.82, p = 0.78). In the subgroup of patients with ICH, there was also no association between TIBC and ICH volume (β = −0.19, p = 0.26). Reasons for iron studies are not significantly different between the ruptured and unruptured groups (Supplemental Table 2) but there was a trend towards fewer patients with workup of anemia in the unruptured group (86% vs 93%). Evaluation of the mean corpuscular volume (MCV) in patients with ruptured vs unruptured aneurysms showed no difference between the two groups (ruptured 85.85 fL (SD 8.44) vs unruptured 87.21 fL (SD 7.26), p = 0.13).

**Table 1:** Patient characteristics stratified by TIBC values within one year of diagnosis.

Variables	All N = 366 (%)	Missing	Low TIBC < 2.20 10^−3^ g/L N = 95 (%)	Non-low TIBC ≥ 2.20 10^−3^ g/L N = 271 (%)	P-value
Female	297 (81.1)	0	72 (75.8)	225 (83.0)	0.12
White race	254 (69.3)	0	74 (77.9)	180 (66.4)	0.04
Black race	48 (13.1)	0	6 (6.3)	42 (15.5)	0.02
Hispanic race	33 (9.0)	0	6 (6.3)	27 (10.0)	0.28
Asian race	9 (2.5)	0	1 (1.1)	8 (3.0)	0.31
Other/unknown race	22 (6.0)	0	8 (8.4)	14 (5.2)	0.26
Age at diagnosis, mean (SD)	57.4 (13.7)	0	59.6 (13.4)	56.5 (13.8)	0.06
Coronary artery disease	32 (8.7)	0	8 (8.4)	24 (8.9)	0.88
Myocardial infarction	24 (6.6)	0	5 (5.3)	19 (7.0)	0.57
Hypertension	196 (53.6)	0	52 (54.7)	144 (53.1)	0.78
Atrial fibrillation	17 (4.6)	0	4 (4.2)	13 (4.8)	0.81
Number of aneurysms, mean (SD)	1.36 (0.80)	0	1.32 (0.72)	1.38 (0.82)	0.49
Family history aneurysms	43 (11.7)	0	6 (6.3)	37 (13.7)	0.05
Current tobacco use	106 (29.0)	4	29 (30.5)	77 (28.4)	0.54
Current alcohol use	138 (37.7)	16	36 (37.9)	102 (37.6)	0.63
Ruptured aneurysms	151 (41.3)	0	69 (72.6)	82 (30.3)	<0.01
Iron (10^−3^ g/L), mean (SD)	0.59 (0.41)	0	0.40 (0.33)	0.66 (0.41)	<0.01
Ferritin (10^−4^ g/L), mean (SD)	2.39 (3.48)	0	5.09 (5.26)	1.45 (1.83)	<0.01

SD = standard deviation.

**Table 2:** Iron, ferritin and TIBC values in the short-term (<1 year after diagnosis) and in the long-term (1–3 years from diagnosis) stratified by rupture status.

	Ruptured Mean (95%CI)	Non-ruptured Mean (95% CI)	P-value
**<1 year**			
Iron (10^−3^ g/L)	0.49 (0.43–0.56)	0.65 (0.60–0.71)	<0.01
Ferritin (10^−4^ g/L)	3.08 (2.43–3.73)	1.91 (1.51–2.30)	<0.01
TIBC (10^−3^ g/L)	2.47 (2.33–2.61)	3.02 (2.93–3.12)	<0.01
Albumin (g/dL)	3.87 (0.51)	3.88 (0.57)	0.84
**1–3 years**			
Iron (10^−3^ g/L)	0.65 (0.49–0.80)	0.67 (0.61–0.72)	0.75
Ferritin (10^−4^ g/L)	1.86 (1.17–2.55)	1.52 (1.12–1.92)	0.43
TIBC (10^−3^ g/L)	2.88 (2.54–3.22)	3.11 (3.00–3.22)	0.11
Albumin (g/dL)	4.02 (3.84–4.21)	4.04 (3.95–4.13)	0.88

CI = confidence interval.

Table 3 shows the results of the univariable and multivariable analyses. In univariable analysis, younger age (OR 0.97, 95% CI 0.96–0.99), other/unknown race (OR 2.61, 95% CI 1.06–6.44), current tobacco use (OR 1.93, 95% CI 1.22–3.06), and current alcohol use (OR 2.12, 95% CI 1.36–3.29) were significantly associated with aneurysm rupture. In contrast, coronary artery disease (OR 0.37, 95% CI 0.16–0.88), myocardial infarction (OR 0.35, 95% CI 0.13–0.97), family history of aneurysms (OR 0.45, 95% CI 0.22–0.92), lower iron (OR 0.33, 95% CI 0.18–0.60), ferritin (OR 1.11, 95% CI 1.04–1.19), and lower TIBC values (OR 0.39, 95% CI 0.29–0.53) within 1 year of diagnosis were significantly and inversely associated with intracranial aneurysm rupture.

**Table 3:** Univariable and multivariable logistic regression for rupture status including iron related laboratory values within 1 year of diagnosis (N = 366).

Characteristics	Univariable		Multivariable		Multivariable*	
	OR (95% CI)	P-val.	OR (95% CI)	P-val.	OR (95% CI)	P-val.
Female	0.89 (0.53–1.52)	0.68	—	—	—	—
Black race (vs. white race)	0.75 (0.39–1.43)	0.38	1.08 (0.51–2.32)	0.84	1.00 (0.47–2.12)	0.99
Hispanic race (vs. white race)	1.24 (0.60–2.58)	0.56	1.56 (0.67–3.62)	0.30	1.43 (0.62–3.29)	0.40
Asian race (vs. white race)	1.19 (0.31–4.55)	0.80	2.13 (0.47–9.69)	0.33	1.84 (0.41–8.36)	0.43
Other/unknown race (vs. white race)	2.61 (1.06–6.44)	0.04	3.91 (1.23–12.43)	0.02	3.41 (1.16–10.04)	0.03
Age at diagnosis	0.97 (0.96–0.99)	<0.01	0.97 (0.95–0.99)	<0.01	0.97 (0.95–0.99)	<0.01
Coronary artery disease	0.37 (0.16–0.88)	0.02	—	—		
Myocardial infarction	0.35 (0.13–0.97)	0.04	—	—		
Hypertension	0.77 (0.50–1.16)	0.21	—	—		
Atrial fibrillation	0.42 (0.14–1.32)	0.14	—	—		
Number of aneurysms	1.15 (0.89–1.50)	0.28	—	—		
Family history aneurysms	0.45 (0.22–0.92)	0.03	0.48 (0.21–1.07)	0.07	0.45 (0.20–1.01)	0.05
Current tobacco use (vs. not current)	1.93 (1.22–3.06)	<0.01	1.63 (0.95–2.82)	0.08	1.45 (0.85–2.48)	0.17
Current alcohol use (vs. not current)	2.12 (1.36–3.29)	<0.01	2.65 (1.56–4.48)	<0.01	2.58 (1.52–4.37)	<0.01
Iron (10^−3^ g/L)	0.33 (0.18–0.60)	<0.01	0.52 (0.27–1.00)	0.053	0.52 (0.27–0.99)	0.05
Ferritin (10^−4^ g/L)	1.11 (1.04–1.19)	<0.01	1.02 (0.94–1.10)	0.68	1.01 (0.93–1.10)	0.77
TIBC (10^−3^ g/L)	0.39 (0.29–0.53)	<0.01	0.41 (0.28–0.60)	<0.01	0.41 (0.28–0.59)	<0.01

Multiple imputation (40 imputations) with chained equations was used for missing data.

*Complete cases only (N = 349).

**Figure 1:**
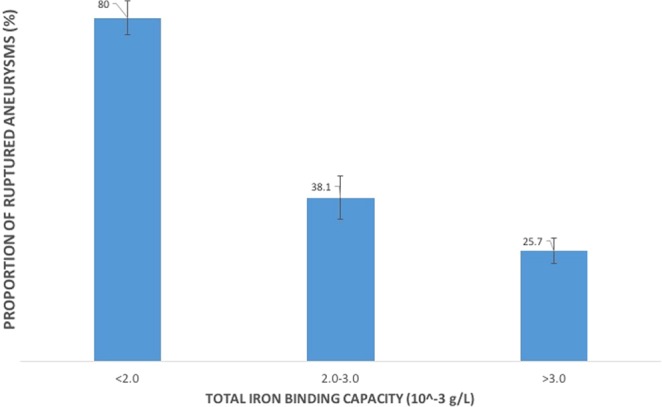
Percentage of ruptured aneurysms stratified according to mean TIBC values.

In multivariable analysis, younger age (OR 0.97, 95% CI 0.95–0.99), other/unknown race (OR 3.91, 95% CI 1.23–12.43), and current alcohol use (OR 2.65, 95% CI 1.56–4.48) were significantly associated with aneurysmal subarachnoid hemorrhage. In contrast, TIBC within 1 year of diagnosis was significantly inversely associated with ruptured aneurysms (OR 0.41, 95% CI 0.28–0.60). The direction and significance of all coefficients remained the same in the sensitivity analyses using complete cases only (Table 3). TIBC was also significantly and inversely associated with rupture in patients with available long-term TIBC values (OR 0.52, 95% CI 0.29–0.93) (Supplemental Table 3). Figure 1 shows the proportion of ruptured aneurysms stratified according to TIBC levels.

## Discussion

In the present study, we examined the association between ruptured intracranial aneurysms and serum iron, ferritin, and total iron binding capacity (TIBC) in the short-term (<1 year) and long-term (between 1 and 3 years) period from diagnosis. We demonstrated that while serum iron and ferritin were not significant in multivariable analyses, low TIBC appeared to be a significant predictor of rupture status in both the short-term and long-term. One should note, however, that there may be a selection bias as only a subset of patients underwent iron studies.

Although serum iron was significantly lower, and serum ferritin was significantly higher in patients with ruptured aneurysms in the short-term, which was reflected in the univariable analysis, this significance disappeared when controlled for confounders in short-term multivariable analysis and in all long-term (univariable and multivariable) analyses. This suggests acute phase effects of these parameters in response to aSAH, rather than a reflection of low (iron) or high (ferritin) baseline values. Indeed, serum ferritin is a well-known acute phase reactant, which can occur in response to any inflammatory or infectious process^19^. In contrast, multivariable analysis showed TIBC values between 1 and 3 years from diagnosis as an independent predictor of rupture status. Although transferrin (and its indirect measure, TIBC) are also considered (negative) acute phase reactants, our long-term findings suggest TIBC values to be a reflection of low baseline values, rather than a negative acute phase response^19,20^. Since low transferrin and TIBC could also be markers of malnutrition^21–23^, which is a common finding among survivors of aSAH^24,25^, our results could be explained by impaired nutritional status in the setting of post aneurysmal subarachnoid hemorrhage. However, Sergi *et al*. compared 44 underweight patients with 69 normal or overweight subjects, and found no correlation between transferrin and fat-free mass^26^. In contrast, albumin levels were significantly lower in underweight subjects, and this finding was confirmed by other authors, making albumin a stronger biomarker of malnutrition, particularly in the setting of surgical outcomes^26–28^. Therefore, in order to control for this possible bias, we compared albumin values between ruptured and non-ruptured patients, and found no statistical significance, making malnutrition an unlikely cause for lower TIBC values among ruptured patients. Low TIBC could also be explained by liver disorders, however, the lack of significant difference in albumin, an important measure of liver function and a measure of liver cirrhosis, between the low and high TIBC groups makes liver disorders an unlikely cause for lower TIBC values among ruptured patients. Moreover, there is no difference in alcohol use between the low and high TIBC groups, making differences in alcoholic liver disease between the two groups less likely.

Low transferrin values can be found in a variety of pathologies, including anemia of chronic disease, malignancy, infection, and nephrotic syndrome. In a prospective cohort of 807 patients with maintenance hemodialysis, patients with lower TIBC levels (<250 μg/dL) had higher markers of inflammation, including interleukin-6 (IL-6) and serum C-reactive protein (CRP) values^21^. Indeed, it has been shown that IL-6 has a negative effect on transferrin synthesis, both *in vitro*^29^ and *in vivo*^30^. Kobune *et al*. showed that in rats, IL-6 administration led to a decrease of total iron binding capacity after 6 hours, confirming the mechanistic association between this cytokine and TIBC^30^. Interestingly, Cheuk *et al*. evaluated the secretion of IL-6 in explant cultures of human abdominal aneurysm biopsies and found IL-6 secretion to be significantly higher in ruptured aneurysms than in intact aneurysms, suggesting the role of IL-6 in abdominal aneurysm rupture^31^. IL-6 has also been investigated in the context of intracranial aneurysms. Morgan *et al*. showed that two different haplotypes (572 C/174 G) which are associated with increased synthesis of IL-6, were statistically associated with increased intracranial aneurysm susceptibility^32^, and in a recent meta-analysis the association between the G572C polymorphism and ruptured and unruptured aneurysms was shown to be significant^33^.

Another biomarker that has been associated with lower TIBC values is increased C-reactive protein (CRP)^21^, which has previously emerged as a risk factor for arteriosclerosis and cardiovascular disease, and as a predictor of myocardial infarctions and stroke^21^. Interestingly, CRP has also been associated with abdominal aortic aneurysm pathophysiology. De Haro *et al*. showed a significant association between high-sensitive CRP values and asymptomatic aortic aneurysm expansion, which reflects the magnitude of the degenerative process of the aneurysm wall and functions as a surrogate marker of rupture risk^34^. Taken together, our findings support the hypothesis that both short-term and long-term TIBC are significantly associated with ruptured intracranial aneurysms, possibly under influence of pro-inflammatory cytokines and CRP that play a role in both acute and chronic inflammation and aneurysm wall degradation.

One of the major strengths of our study is the high-quality standardized database, a control group with unruptured intracranial aneurysms, and the availability of important risk factors, such as smoking status, alcohol intake, and hypertension. The main limitations of our study include the small subgroup of mainly anemic patients with available iron-related parameters, the retrospective design for a portion of the patients, and the lack of pre-rupture measurements of iron, ferritin, and TIBC in patients with ruptured aneurysms. In addition, the proportion of patients with ruptured aneurysms in the entire cohort was much less than in those who underwent iron studies (27.7% vs 41.3%). While we did not find a significant difference in the reasons for the iron studies being done, it is still possible that there was a selection bias. Hence the associations found in this study may not be generalizable to the general population with aneurysms. The lack of liver function tests is another limitation, since serum iron parameters could be affected by liver disorders. Finally, in some cases of aSAH, history of tobacco and alcohol consumption were not obtained directly from patients in poor clinical conditions, which could have led to information bias.

## Conclusions

Our data show that low total iron binding capacity is significantly associated with aneurysmal subarachnoid hemorrhage. However, one must use caution in interpreting this association as the data only included patients who underwent iron studies so that there may be a selection bias. Further prospective trials are needed to confirm our findings.

## Supplementary information

Supplemental File

## Data Availability

The datasets generated during and/or analysed during the current study are available from the corresponding author on reasonable request.
