# Supplementary material for: Decreased Total Iron Binding Capacity May Correlate with Ruptured Intracranial Aneurysms
Source: Sci Rep. 2019 Apr 15;9:6054. doi: 10.1038/s41598-019-42622-y (PMC6465340; doi:10.1038/s41598-019-42622-y)
Supplement: Supplementary file 1 — Supplemental File [file 41598_2019_42622_MOESM1_ESM.docx]

**Decreased Total Iron Binding Capacity May Correlate with Ruptured Intracranial Aneurysms**

Anil Can, MD^1^, Pui Man Rosalind Lai, MD^1^, Victor M. Castro, MS^2^, Sheng Yu, PhD^3^, Dmitriy Dligach, PhD^4^, Sean Finan, BS^5^, Vivian Gainer, MS^2^, Nancy A. Shadick, MD, MPH^6^, Guergana Savova, PhD^5^, Shawn Murphy, MD, PhD^2,7^, Tianxi Cai, PhD^8^, Scott T. Weiss, MD, MS^9^, Rose Du MD, PhD^1,9^*

^1^Department of Neurosurgery, Brigham and Women’s Hospital, Harvard Medical School, Boston, MA, USA

^2^Research Information Systems and Computing, Partners Healthcare, Boston, MA

^3^Center for Statistical Science, Tsinghua University, Beijing, China

^4^Department of Computer Science, Loyola University, Chicago, IL

^5^Boston Children’s Hospital Informatics Program, Boston, MA

^6^Division of Rheumatology, Immunology and Allergy, Brigham and Women’s Hospital, Boston, MA

^7^Department of Neurology, Massachusetts General Hospital, Boston, MA

^8^Biostatistics, Harvard School T. H. Chan of Public Health, Boston, MA

^9^Channing Division of Network Medicine, Brigham and Women’s Hospital, Boston, MA

***Corresponding author**

Rose Du, M.D., Ph.D.

Department of Neurosurgery

Brigham and Women’s Hospital

75 Francis Street

Boston, MA 02115

Phone: 617-732-6600

Fax: 617-734-8342

Email: rdu@bwh.harvard.edu

**Supplemental Table 1.** Iron, ferritin and TIBC values (<1 year after diagnosis) stratified by sex.

|  | **Male**  **Mean (SD)** | **Female**  **Mean (SD)** | **P-value** |
| --- | --- | --- | --- |
| Iron (10^-3^ g/L) | 0.59 (0.39) | 0.59 (0.41) | 0.52 |
| Ferritin (10^-4^ g/L) | 3.98 (5.36) | 2.04 (2.78) | <0.01 |
| TIBC (10^-3^ g/L) | 2.58 (0.81) | 2.84 (0.80) | 0.99 |

**Supplemental Table 2.** Reasons for iron studies stratified according to rupture status.

| **Reason for iron studies** | **N** | **Unruptured N=217** | **Ruptured N=149** | **P-value** |
| --- | --- | --- | --- | --- |
| Anemia | 324 | 186 | 138 | 0.06 |
| Abnormal LFTs/liver disease | 8 | 6 | 2 | 0.58 |
| Gastrointestinal bleed | 3 | 3 | 0 | 0.39 |
| Restless leg syndrome | 3 | 2 | 1 | 1 |
| Vitamin D deficiency | 3 | 2 | 1 | 1 |
| Gastric bypass | 2 | 2 | 0 | 0.65 |
| Hemachromatosis | 2 | 2 | 0 | 0.65 |
| Hypercoagulability workup | 2 | 1 | 1 | 1 |
| Alcohol abuse | 2 | 0 | 2 | 0.32 |
| Other/unknown* | 17 | 11 | 5 | 0.60 |

***** Other includes 3 unknown reasons, and 1 of each of the following: antiphospholipid syndrome, sickle cell trait, hematemesis, uterine fibroids, hyperthyroidism, systemic lupus erythematosus, chronic kidney disease, leukocytosis, Crohn’s disease, persistent diarrhea, neuropathy, hair loss, fatigue, dizziness.

LFT = liver function test

**Supplemental Table 3.** Univariable and multivariable logistic regression for rupture status including iron related laboratory values between 1 and 3 years around diagnosis (N=200).

|  | **Univariable** | | **Multivariable** | |
| --- | --- | --- | --- | --- |
| **Characteristics** | **OR (95% CI)** | **P-val.** | **OR (95% CI)** | **P-value** |
| Female | 0.71 (0.32-1.60) | <0.01 | - | - |
| Black race (vs. white race) | 2.47 (0.86-7.13) | 0.09 | **2.62 (0.79-8.72)** | 0.12 |
| Hispanic race (vs. white race) | 5.77 (2.30-14.48) | <0.01 | 7.61 (2.64-21.93) | <0.01 |
| Other/unknown race (vs. white race) | 1.65 (0.32-8.46) | 0.55 | 1.55 (0.26-9.14) | 0.63 |
| Age at diagnosis | 0.95 (0.93-0.98) | <0.01 | 0.95 (0.92-0.98) | <0.01 |
| Coronary artery disease | 0.41 (0.09-1.83) | 0.24 | - | - |
| Myocardial infarction | 0.58 (0.13-2.70) | 0.49 | - | - |
| Hypertension | 1.39 (0.69-2.78) | 0.36 | - | - |
| Atrial fibrillation | 1.63 (0.49-5.49) | 0.43 | - | - |
| Number of aneurysms | 1.33 (0.88-1.99) | 0.17 | - | - |
| Family history aneurysms | 1.32 (0.55-3.18) | 0.54 | 1.05 (0.37-2.95) | 0.93 |
| Current tobacco use (vs. not current) | 2.61 (1.23-5.50) | 0.01 | 1.76 (0.72-4.32) | 0.22 |
| Current alcohol use (vs. not current) | 2.68 (1.33-5.38) | <0.01 | 2.14 (0.93-4.93) | 0.08 |
| Iron (10^-3^ g/L) | 0.87 (0.36-2.10) | 0.75 | 0.90 (0.33-2.48) | 0.84 |
| Ferritin (10^-4^ g/L) | 1.05 (0.93-1.18) | 0.44 | 0.96 (0.80-1.14) | 0.62 |
| TIBC (10^-3^ g/L) | 0.72 (0.47-1.08) | 0.11 | 0.52 (0.29-0.93) | 0.03 |

CI = confidence interval
